# Draft Genome Sequences of *Pseudomonas aeruginosa* Strain PS3 and *Citrobacter freundii* Strain SA79 Obtained from a Wound Dressing-Associated Biofilm

**DOI:** 10.1128/genomeA.00561-15

**Published:** 2015-06-04

**Authors:** Sirwan Akbar, Simon P. Rout, Paul N. Humphreys

**Affiliations:** Department of Biological Sciences, School of Applied Sciences, University of Huddersfield, Queens Gate, Huddersfield, United Kingdom

## Abstract

Two isolates, one from the genus *Pseudomonas* and the second from *Citrobacter*, were isolated from a wound dressing-associated biofilm. Following whole-genome sequencing, the two isolates presented genes encoding for resistance to antibiotics and those involved in exopolysaccharide production.

## GENOME ANNOUNCEMENT

The management of infected wounds is a worldwide health care issue (1), exacerbated by the rise of antimicrobial resistance (2–4). The formation of biofilms within wounds has been implicated in the delayed healing, ineffective treatment, and prolonged infection of chronic wounds (5) through the suppression of the immune system and the reduced impact of antimicrobial agents (5–8). *Pseudomonas* spp. have previously been identified as both a pathogen and a biofilm-forming organism within a wound environment, particularly in those wounds associated with burns (9, 10). In contrast, instances of *Citrobacter* sp. wound infections are uncommon within the literature; however, there are reported instances of their pathogenicity (11, 12). Here, we present the draft genome sequences of *Pseudomonas aeruginosa* strain PS3 and *Citrobacter freundii* strain SA79, both of which were isolated from a contaminated wound dressing.

A discarded dressing from an infected wound was provided anonymously from a local skin integrity practitioner. Swabs were taken from the wound surface and transferred to 2 mL of sterile maximum recovery diluent (LabM Ltd.) and vortexed. The homogeneous suspension was then used to prepare 400-µL spread plates upon a *Pseudomonas aeruginosa* selective medium (LAB108, with X107 supplement, LabM, United Kingdom). Single colonies were selected from the plate and purified through further subculture before total genomic DNA was isolated using a commercial kit (Ultraclean Microbial Isolation Kit, Mo-Bio, USA).

Draft whole-genome sequences were obtained using a whole-genome shotgun (WGS) sequence strategy. Paired-end 125 cycles sequence reads were generated using the Illumina HiSeq 2500 system (BaseClear, Netherlands). FASTQ sequence files were generated using the Illumina Casava pipeline version 1.8.3 and the assembly prepared using CLC Genomics Workbench version 7.0.4. The contigs were linked and placed into scaffolds or supercontigs. The orientation, order, and distance between the contigs was estimated using the insert size between the paired-end and/or mate-pair reads using the SSPACE Premium scaffolder version 2.3 (13). The draft genome sequencing of *Pseudomonas aeruginosa* strain PS3 generated 165 contigs, with a sequence length of 6,799,547 bp (66.2% G+C content). The draft genome contained a total of 6,161 coding sequences (CDSs), where 35 pseudogenes, 2 genes coding for rRNA (16S, 23S), 57 genes coding for tRNA, and 1 noncoding RNA (ncRNA) were present. *Citrobacter freundii* strain SA79 was 4,870,483 bp in length across 19 contigs with a G+C content of 51.7%. The draft genome contained a total of 4,480 CDSs, 36 pseudogenes, 3 genes coding for rRNA (5S, 16S, 23S), 72 genes coding for tRNA, and 9 ncRNAs were present. Further analysis of the two genomes using RAST (14) indicated that both organisms carried genes encoding resistance to antibiotics and toxic compounds. In addition, the presence of genes involved with exopolysaccharide and biofilm synthesis suggests that these organisms may be of further clinical interest.

### Nucleotide sequence accession numbers. 

These sequences were submitted to Genbank under the accession numbers JRGP00000000 (*Pseudomonas aeruginosa* strain PS3) and LAZI00000000 (*Citrobacter freundii* strain SA79).
